# Influence of HLA class I, HLA class II and KIRs on vertical transmission and chronicity of hepatitis C virus in children

**DOI:** 10.1371/journal.pone.0172527

**Published:** 2017-02-22

**Authors:** A. Ruiz-Extremera, E. J. Pavón-Castillero, M. Florido, P. Muñoz de Rueda, J. A. Muñoz-Gámez, J. Casado, A. Carazo, R. Quiles, S. M. Jiménez-Ruiz, A. Gila, J. D. Luna, J. León, J. Salmerón

**Affiliations:** 1 Paediatric Unit, San Cecilio University Hospital and Virgen de las Nieves University Hospital, Granada, Spain; 2 Paediatric Department, Granada University, Granada, Spain; 3 CIBER for Liver and Digestive Disease (CIBERehd), Instituto de Salud Carlos III, Spain; 4 Instituto de Investigación Biosanitaria de Granada, Spain; 5 Clinical Management Unit of Digestive Diseases, Research Unit, San Cecilio University Hospital, Granada, Spain; 6 Medicine Department, Granada University, Granada, Spain; 7 Biostatistic Department, Granada University, Granada, Spain; University of North Carolina at Chapel Hill School of Dentistry, UNITED STATES

## Abstract

**Background & aim:**

There is evidence that maternal viral load of HCV during delivery influences the risk for Mother-to-child transmission (MTCT), but this does not explain all cases. We study the role of the immunogenetic profile (HLA, KIRs and KIR-ligand binding) of mothers and children in HCV-MTCT and in chronicity in the children.

**Methodology:**

**7**9 HCV-RNA (+) mothers and their 98 children were included. 24 children were infected, becoming chronic in 8 cases and clearing in 16. HLA-class-I and II and KIRs were determined by Luminex.

**Results:**

MTCT study: The presence of HLA-C1-ligand in mothers and/or their children reduces the risk of transmission (mothers: Pc = 0.011, children: P = 0.033), whereas the presence of HLA-C2C2-ligand in mothers increases it (Pc = 0.011). In children KIR2DL3-HLA-C1 is a protector factor (Pc = 0.011). Chronicity in children study: Maternal DQA1*01 allele (Pc = 0.027), KIR2DS1 (Pc = 0.011) or KIR3DS1 (Pc = 0.011) favours chronicity in the child. The presence of the DQB1*03 allele (Pc = 0.027) and KIR2DS3 (P = 0.056) in the child and homozygosity for KIR3DL1/3DL1 (Pc = 0.011) and for the HLA-Bw4/Bw4 ligand (P = 0.027) is associated with viral clearance, whereas the presence of HLA-Bw6 ligand (P = 0.027), the binding of KIR3DS1-HLA-Bw4 (P = 0.037) and heterozygosity for KIR3DL1/3DS1 (Pc = 0.011) favour viral chronicity. Mother/child allele matching: In the joint HLA analysis, matching was greater between mothers and children with chronic infection vs those who had cleared the virus (67%±4.1 vs 57%±1.2, P = 0.003).

**Conclusions:**

The HLA-C1 ligand in the mother is related to MTCT, while several genetic factors of the mother or child are involved in the chronification or clearance of infection in the child. Matching allelic data is considered to be an indicator of HCV chronicity in the child and can be used as a potential prognostic test. This implies that NK cells may play a previously undocumented role in protecting against MTCT and that both NK cell immunity and adaptive T-cell responses may influence viral clearance in infected children.

## Introduction

Hepatitis C virus (HCV) is a major health problem, affecting 170 million people worldwide. The prevalence in the United States and Europe is 1–2%, but it can reach 8% in some developing countries[1,2] and 0.05–5% in children[3]. In Spain, the prevalence in pregnant women is 0.5%-1.4%[4,5], similar to that of the general population; however, this prevalence may have changed due to the increase in the immigrant population over recent years. Mother-to-child transmission (MTCT) is the major cause of paediatric HCV infection, which is the most common origin of chronic liver disease in children in industrialised countries. The pathogenesis of HCV during pregnancy and the neonatal period remains poorly understood. The MTCT rate of HCV is 1–8% in mothers who are not co-infected with HIV and around 20% in co-infected mothers. 90% of infected children acquire the virus by vertical transmission. Children can spontaneously clear the virus during the first few months of life, but 53% of those who persist with intermittent viraemia remain chronically infected[6–8]. However, MTCT is not observed in all mothers with high viral load and can occur in mothers with low viral load in the absence of HIV co-infection, suggesting that other factors are involved. One explanation for the low MTCT rate is the small amount of inoculum that the child receives from the mother. This would explain why the viral load at the time of birth is an important predictive factor. Nevertheless, despite extensive research, to date the only factors for which there is adequate scientific evidence related to increased MTCT risk are viral load and HIV co-infection. A vigorous response by cytotoxic T-cells during acute HCV infection increases the lysis of infected hepatocytes, decreases virion production and reduces the probability of infection chronification[9]. Current genetic-immunological studies suggest that host genetic factors influence the response to antiviral treatment and spontaneous HCV clearance in adults. Few studies have been conducted on the influence of HLA genes on MTCT rate and chronicity or spontaneous clearance in infected children. The immune response and HLA class I and HLA class II may be important determinants of HCV outcomes[10]. It is therefore important to perform a complete study of HLA alleles and other genetic factors, such as killer-cell immunoglobulin-like receptors (KIRs), in the mother and the child. Natural killer (NK) cell function is regulated by the fine balance of cell surface inhibitory and activating receptors, including C-type lectin receptors and KIRs[11,12].

As so few studies of these genetic factors have been published, we carried out a comprehensive study of HLA, KIRs and binding KIR-ligand-HLA. The main aim of this research is to improve our understanding of the factors that influence HCV-MTCT with particular reference to the phenomena of spontaneous viral clearance and chronicity in children.

## Materials and methods

### Subjects

A prospective cohort study was conducted between 1991 and 2009 in consecutive mothers routinely tested for HCV during prenatal care at San Cecilio University Hospital in Granada (Spain). The study included 79 HCV-RNA-positive mothers with their 98 children, who were followed up for at least 6 years. The study population is the same mother-child cohort as described in an earlier article, although with fewer patients due to the absence of plasma samples [7]. All participants were Caucasian. Exclusion criteria were the presence of hepatitis B virus, HIV, alcoholism or autoimmune disease. The mothers were tested for the study parameters during the final trimester of pregnancy (from week 28 of gestation to the end of the pregnancy), at delivery and during the post-partum period. The diagnosis of MTCT was based on detectable HCV-RNA in the peripheral blood by PCR. MTCT was defined as children who presented HCV-RNA (+) in at least two subsequent blood samples. The study groups for MTCT were: (1) transient viraemia, infants who exhibited HCVRNA (+) in at least two subsequent blood samples with posterior HCV-RNA (-) and without serum-conversion; (2) chronic or persistent infection group, defined as children with persistent HCV-RNA (+) with HCV serum-conversion (detectable anti-HCV). The criterion of HCV-RNA(+) in at least two samples was established to minimise the risk of false positives. When the infants presented an initial HCV-RNA (+) test, a further analysis was performed in a new blood sample, a few days later, in order to confirm the first positive and to determine the viral genotype. No false positives were recorded in this study and all infants were HCVRNA (+) in the second test.

Data were compiled for HCV-MTCT risk factors, transient viraemia, chronic infection, HCV viral load, genotype, delivery mode, duration of ruptured membranes, ALT levels, breastfeeding yes/no and duration of breastfeeding. The infants were examined by paediatricians and tested for HCV-RNA at birth, at 2, 4, 6, 8, 10, 12, 18 and 24 months and then at 3, 4, 5 and 6 years.

### Ethical considerations

Informed written consent was obtained in all cases (from each mother and by them on behalf of their children), for the collection and storage of serum and peripheral blood for DNA extraction. The study complied with the ethical guidelines of the 1975 Helsinki Declaration, as reflected in the a priori approval granted by the San Cecilio University Hospital Ethics Committee (Granada, Spain).

### Virological assays

Reverse hybridisation was used for HCV genotyping (Inno-LIPA II HCV Innogenetics SA Ghent, Belgium). Viral load (cut-off <15 IU/mL) was quantitatively measured in serum with the HCV AmpliprepTaq-Man, Roche Molecular System, at the same time as the ALT determination.

### HLA class I, HLA class II and KIR allele typing

Genotyping of class I HLA-A, B, and Cw loci, class II HLA-DRB1, DQA1, DQB1, DPA1 and DPB1 loci and KIRs was performed in 177 subjects (79 mothers and 98 children). HLA genotyping was performed using the LABType SSO Genotyping PCR kit (One Lambda, Canoga Park, CA). PCR amplification of target DNA was conducted with sequence-specific primers, followed by hybridisation with allele-specific oligodeoxynucleotides coupled with fluorescent phycoerythrin-labelled microspheres. Fluorescence intensity was determined on a Bio-Plex200 system (Luminex xMAP, Austin, TX). HLA-tools software (Los Angeles, CA) was used for HLA allele assignment. KIR typing was performed for 14 KIR genes and two pseudo-KIR genes by the reverse sequence-specific oligonucleotide (RSSO) method, using the LABType SSO Genotyping PCR kit (One Lambda Inc., Canoga Park, CA, USA). Among these, 8 KIR genes (2DLI, 2DL2, 2DL3, 2DL4, 2DL5, 3DL1, 3DL2 and 3DL3) were found to be responsible for inhibitory functions and 6 KIR genes (2DS1, 2DS2, 2DS3, 2DS4, 2DS5, and 3DS1) conveyed activating signals. Oligonucleotide probe-coated beads were utilised for hybridisation with the PCR amplicons covering exons 3, 4, 5, 7, 8, and 9 on chromosome 19. Quantitative data were obtained using the Luminex-200 system (Luminex Corporation, Austin, TX) and were analysed with Luminex 100 ™ IS v2.3 software, following the manufacturer’s instructions. Based on the number and type of KIR genes, two broad haplotype groups were defined: Group-A haplotypes with a fixed KIR gene content of KIR2DL1, -2DL3, -2DL4, -3DL1, -3DL2, -3DL3, -2DS4 and pseudogenes KIR2DP1 and KIR3DP1. Haplotypes carrying any other combination of KIRs were classified as Group B. In general, Group B haplotypes have a more strongly activated KIR gene profile, while Group A ones have a more inhibited KIR gene profile, with KIR2DS4 as the only activating KIR gene. The Group B haplotypes were collectively termed Bx, since they represent a mixture of AB and BB haplotypes. The KIR genotype profiles were assigned to the AA and Bx haplotype groups using the New Allele Frequency Database: http://www.allelefrequencies.net[13].

### Statistical analysis

The dependent variables were MTCT and the degree of HCV chronic infection among the infants (both variables are binary). The measures for the children are nested in those of the mothers, and so independence of these values cannot be considered. Accordingly, a generalised estimating equation (GEE) model was used in each analysis; the models were specified for a binomial family and for the natural link in this case, namely the logit. Due to the small sample size available and the sparsity of data in some cases, the GEE analysis of the Firth model was used. All bivariate analyses to assess the association of the HLA and KIR markers were adjusted by IL28B and viral load. Multivariate analyses were performed using the same models as the bivariate analysis with the variables that were significant or which tended towards statistical significance. The degree of association of the HLA and KIR markers was computed using the odds ratio (OR) from the adjusted GEE model and its 95% CI.

For the percentage of matching of HLA and KIR markers in mother and child (quantitative variables), the results are expressed as mean and standard error of the mean and the analyses were performed using the previously cited GEE model but with family normal and the natural link in this case, identity; logarithm, square root or Box-Cox transformations were used as necessary to obtain normality.

A very large number of comparisons were made between two groups (transmitted and non-transmitted HCV, and between chronically and non-chronically infected), and therefore some precautions regarding significance were necessary. Bonferroni’s correction, which is commonly employed, was not used in this case due to its conservative bias and the diminished power of the results obtained. Instead, a modification of the Benjamini-Yekutieli method to control the false discovery rate was applied.

The area under the ROC curve (AUC) was used to measure the predictive performance for chronicity of the infection in the child according to the mother-child matching in HLA markers. This AUC was computed using the binomial model. Due to the small sample size of chronically ill children, the AUC varied widely.

Data management and analysis were performed using SPSS v19 for Windows (SPSS Inc., Chicago, IL) and STATA 14.1 and its xtgee commands (StataCorp. 2015. Stata Statistical Software: Release 14. College Station, TX: StataCorp LP).

## Results

### 1. Characteristics of mothers and children

The study included 79 mothers who were HCV-RNA(+) and 98 children, including one set of twins and 18 mothers with multiple deliveries. Therefore, we studied 98 mother/child cases. MTCT was observed in 24 of the 98 (24.4%) HCV-RNA(+) cases. Among the 24 children who were infected, the disease became chronic in 8 (33.3%; 8/98, 8.1%) and the virus was cleared in 16 (66.6%). The mean age of the mothers was 28.7±0.5 years. 75% (n = 71) were HCV genotype 1 and the viral load was >600,000 UI/mL in 56%. 57% (n = 56) of the children were male. The mean birth weight was 3,142±57 grams. 15 (16%) children were born by Caesarean section and 68% (n = 66) were breastfed, for an average of 80±10 days.

### 2. Mother to child transmission of HCV

#### 2.1. HLA class I, class II and KIR alleles

Genomic typing (high and low resolution) was performed for class I and class II loci, and all 14 KIR genes and the two pseudogenes were determined in both study groups. In this study, both MTCT and chronicity were based on the number of pregnancies of HCV-RNA(+) mothers (n = 98), not on the number of mothers. Genetic variability in HLA class I and class II is shown in S1 Table, and the mean values of the individual frequencies of all the KIR genes/pseudogenes are shown in S2 Table. Table 1 shows the high and low-resolution HLA of the mothers and children. The differences are statistically significant for MTCT. Only the presence in the mothers of the Cw*06 and Cw*06:02 alleles was associated with MTCT, after application of the Benjamini-Yekutieli correction. In the children, no forms of HLA were related to HCV infection.

**Table 1:** HLA-genomics, high and low resolution typing and KIR genotyping in MTCT of HCV.

	All (n = 98)	Infected (n = 24)	Noninfected (n = 74)	OR^#^	CI	p-value	Pc
**HLA genomics, high resolution typing**							
**Mother, n(%)**							
- B*35:01	11 (11)	6 (25)	5 (7)	5.6	1.5–21.4	0.011	ns
- B*50:01	3 (3)	3 (13)	0 (0)	16.9	0.7–404	0.081	ns
- Cw*06:02	12 (12)	7 (29)	5 (7)	**9.2**	**1.8–55.5**	**0.009**	**0.01**
- DRB1*13:02	10 (10)	5 (21)	5 (7)	5.5	1.1–26.1	0.033	ns
- DQA1*03:01	17 (17)	0 (0)	17 (23)	0.09	0.01–8.5	0.098	ns
**Child, n(%)**							
- DPB1*02:01	26 (26)	11 (46)	15 (20)	2.7	0.9–7.8	0.068	ns
**HLA genomics, low resolution typing**							
**Mother, n(%)**							
- B*50	3 (3)	3 (13)	0 (0)	16.9	0.7–404	0.081	ns
- Cw*06	12 (12)	7 (29)	5 (7)	**9.2**	**1.8–55.5**	**0.009**	**0.01**
- DRB1*04	25 (25)	2 (8)	23 (31)	0.2	0.04–1.1	0.065	ns
- DRB1*13	35 (35)	13 (54)	22 (29)	2.5	0.9–6.91	0.078	ns
- DQA1*03	24 (24)	2 (8)	22 (29)	0.3	0.06–1.3	0.095	ns
**Child (n = 98)**							
- DPB1*02	32 (32)	12 (50)	20 (27)	2.0	0.7–5.6	0.177	ns
**KIR genotyping: children, n(%)**							
- KIR2DL3	93 (95)	20 (83)	73 (99)	0.1	0.01–1.4	0.087	ns

Values are absolute with percentages in parentheses.

MTCT; Mother-to-child transmission, HCV; Hepatitis C virus, HLA; Human leucocyte antigen, KIR; Killer-cell-immunoglobulin-like receptors, OR; Odds ratio, CI; Confidence interval, Pc; Benjamini-Yekutieli correction, ns; Non-significant.

#### 2.2. KIR genes

The presence of KIR2DL3 in the child reflects an increased probability of protection against infection in the child (P<0.1) (Table 1). This pattern remains and is more strongly apparent in the multivariate analysis.

#### 2.3. KIR haplotypes/genotypes

No statistically significant differences were observed among the different haplotype categories A/A and B/x (A/B and B/B) (see S3 Table).

#### 2.4. KIR-HLA associations

2.4.1. KIR-HLA-B: Both KIR3DL1 and KIR3DS1 encode receptors that bind HLA-Bw4 ligands, and are derived from alleles from a single locus. No statistically significant association with MTCT was observed (S3 Table).

2.4.2. KIR-HLA-C: As with the type B alleles, KIR2DL2 and KIR2DL3 are also derived from a single locus and bind to HLA-C1 and HLA-C2 ligands. Table 2 shows the results obtained, and highlights the presence of statistically significant differences. S3 Table shows the results of all the possible combinations studied.

**Table 2:** KIR/HLA-ligands and MTCT of HCV.

**MOTHERS**	**All (n = 98)**	**Infected (n = 24)**	**Noninfected (n = 74)**	**OR**	**CI**	**p-value**	**pc**
-HLA-C1	75 (78)	14 (58)	61 (85)	0.2	0.06–0.7	**0.008**	**0.011**
-HLA-C2	68 (71)	22 (92)	46 (64)	4.3	0.9–20.8	0.074	ns
-HLA-C1C1	28 (29)	2 (8)	26 (36)	0.2	0.05–1.2	0.074	ns
-HLA-C2C2	21 (22)	10 (42)	11 (15)	4.9	1.5–15.9	**0.008**	**0.011**
-KIR2DL2/2DL3-HLA-C2C2	4 (4)	3 (13)	1 (1)	12.2	1.0–150	0.051	ns
-KIR2DL1-HLA-C2	67 (71)	22 (92)	45 (64)	4.2	0.9–20.6	0.078	ns
-KIR2DL3-HLA-C1	67 (70)	13 (54)	54 (75)	0.5	0.2–1.6	0.230	ns
-KIR2DS4-HLA-C1	74 (77)	14 (58)	60 (83)	0.2	0.1–0.7	0.012	ns
-KIR2DS4-HLA-C2	68 (71)	22 (92)	46 (64)	4.3	0.9–20.8	0.074	ns
**CHILDREN**	**All (n = 98)**	**Infected (n = 24)**	**Noninfected (n = 74)**	**OR**^**#**^	**CI**	**p-value**	
-KIR2DL2_2DL2	5 (5)	4 (17)	1 (1)	10.0	0.7–150	0.095	ns
-KIR2DL2_2DL3	46 (47)	7 (29)	39 (52)	0.5	0.1–1.4	0.177	ns
-HLA-C1	81 (84)	17 (71)	64 (89)	0.5	0.1–2.0	0.033	ns
-HLA-C2C2	15 (16)	7 (29)	8 (11)	2.0	0.5–7.7	0.328	ns
-KIR2DL2/2DL2-HLA-C2C2	2 (2)	2 (8)	0 (0)	3.4	0.1–96.8	0.471	ns
-KIR2DL2/2DL3-HLA-C1C2	26 (27)	3 (13)	23 (32)	0.3	0.05–1.3	0.098	ns
-KIR2DL3/2DL3-HLA-C2C2	5 (5)	4 (17)	1 (1)	7.3	0.7–81	0.104	ns
-KIR2DL3-HLA-C1	78 (81)	15 (63)	63 (88)	0.2	0.09–0.7	**0.009**	**0.011**

Values are absolute with percentages in parentheses.

MTCT; Mother-to-child transmission, HCV; Hepatitis C virus, HLA; Human leucocyte antigen, KIR; Killer-cell-immunoglobulin-like receptors, OR; Odds Ratio, CI; Confidence interval, Pc; Benjamini-Yekutieli correction

2.4.2.1. HLA-C1 and HLA-C2 ligand: The presence of the HLA-C1 ligand is associated with a lower risk of infection in children by MTCT (Table 2). Conversely, if the ligand of the mother is of the type HLA-C2C2, there is a greater risk of MTCT (Table 2).

2.4.2.2. Combinations of KIR2DL2 and/or KIR2DL3 with HLA-C1 and/or HLA-C2 ligand. Although all possible associations were analysed, only the presence in the child of the combination KIR2DL3-HLA-C1 was related to a lower risk of MTCT (Table 2).

#### 2.5. Mother/child allele matching and MTCT

Analysis of the rate of mother/child allele matching, for all HLA alleles, class I and II alleles, individual HLA alleles and KIRs, revealed no statistically significant association with MTCT (data not shown).

#### 2.6. Multivariate analysis and the risk of vertical transmission of HCV

Table 3 shows the OR estimated for the logistic regression model, including the genetic factors that are independently associated with MTCT. The factors included in the analysis were the presence of HLA-C1 and HLA-C2C2 ligands in the mother and that of KIR2DL3 in the child. These were the only alleles that could be included, as KIR-HLA combinations had to be excluded, due to data redundancy. The final result shows that both the presence of the HLA-C1 ligand in the mother and that of KIR2DL3 in the child protect against HCV MTCT, independently of other genetic factors.

**Table 3:** Multivariate analysis final model for MTCT of HCV.

	OR	CI	p-value
HLA-C1 ligand mother	0.20	0.05–0.75	**0.018**
HLA-C2C2 ligand mother	1.80	0.32–1.05	0.501
KIR2DL3 child	0.07	0.004–1.14	**0.062**

MTCT: Mother-to-child transmission, HCV; hepatitis C virus, HLA; Human leucocyte antigen, KIR; killer-cell-immunoglobulin-like receptors, OR, Odds Ratio, CI, confidence interval. Multivariate regression analysis.

Reference groups: non-presence HLA-C1 ligands, non-presence HLA-C2C2 ligand, non-presence KIR2DL3 receptor. The OR is the probability of vertical transmission in presence of the allele, with regard to non-presence.

### 3. Chronification of HCV in infected children

#### 3.1. HLA class I, class II and KIR alleles

The infected children of mothers with the DQA1*01 allele had a higher risk of the infection becoming chronic. The presence of the DQB1*03 allele was associated with viral clearance in infected children (Table 4). The children of mothers with KIR2DS1 and KIR3DS1 presented a higher risk of chronicity. Conversely, the presence of KIR2DS3 in the child presented a tendency to viral clearance (Table 4).

**Table 4:** HLA genomics, high and low resolution typing, in chronification of HCV in children.

	All (n = 24)	Chronic (n = 8)	Clearance (n = 16)	OR^#^	CI	p-value	Pc
**HLA genomics, high resolution typing**							
**Mother, n(%)**							
-DQA1*01:01	3 (13)	3 (38)	0 (0)	12.9	0.5–300	0.111	ns
-DQB1*06:04	3 (13)	3 (38)	0 (0)	12.9	0.5–300	0.111	ns
**Child, n(%)**							
-B*08:01	3 (13)	3 (38)	0 (0)	12.9	0.5–300	0.111	ns
-DRB1*03:01	3 (13)	3 (38)	0 (0)	12.9	0.5–300	0.111	ns
-DQA1*05:01	3 (13)	3 (38)	0 (0)	12.9	0.5–300	0.111	ns
-DQB1*02:01	3 (13)	3 (38)	0 (0)	12.9	0.5–300	0.111	ns
**HLA genomics, low resolution typing**							
**Mother, n(%)**							
-DRB1*13	13 (54)	7 (88)	6 (38)	10.2	0.9–118.1	0.062	ns
-DQA1*01	15 (63)	8 (100)	7 (44)	31.3	1.2–857.5	**0.041**	**0.027**
-DQB1*06	13 (54)	7 (88)	6 (38)	10.2	0.9–118.1	0.062	ns
**Child, n(%)**							
-B*08	3 (13)	3 (38)	0 (0)	12.9	0.5–300	0.111	ns
-DRB1*03	3 (13)	3 (38)	0 (0)	12.9	0.5–300	0.111	ns
-DRB1*04	6 (25)	0 (0)	6 (38)	0.09	0.004–2	0.132	ns
-DQA1*03	6 (25)	0 (0)	6 (38)	0.09	0.004–2	0.132	ns
-DQB1*03	13 (54)	1 (13)	12 (75)	0.06	0.005–0.6	**0.019**	**0.027**
**KIR genotyping**							
**Mother, n(%)**							
-KIR2DS1	8 (33)	5 (63)	3 (18)	18.1	1.5–224	**0.024**	**0.011**
-KIR3DS1	9 (38)	6 (75)	3 (19)	20.8	1.7–250	**0.017**	**0.011**
**Child, n(%)**							
-KIR2DS3	7 (29)	0 (0)	7 (44)	0.04	0.002–1.1	0.056	ns

Values are absolute with percentages in parentheses.

HCV; Hepatitis C virus, HLA; Human leucocyte antigen, KIR; Killer-cell immunoglobulin-like-receptors, OR, Odds Ratio, CI, Confidence interval, Pc, Benjamini-Yekutieli correction, ns; Non-significant.

#### 3.2. KIR haplotypes/genotypes

No KIR genotype or haplotype from any of the infected children (n = 24) or from any of their mothers was associated with chronification of HCV in the child (Table 5 and S4 Table).

**Table 5:** Haplotypes/genotypes and chronification of HCV in children.

MOTHERS	All (n = 24)	Chronic (n = 8)	Clearance (n = 16)	OR	CI	p-value	pc
**Haplotypes**							
-A	24 (100)	8 (100)	16 (100)	1	—	—	ns
-B	18 (75)	8 (100)	10 (63)	9.5	0.5–190	0.141	ns
-AA	6 (25)	0 (0)	6 (38)	0.11	0.005–2	0.141	ns
-Bx	18 (75)	8 (100)	10 (63)	9.5	0.5–190	0.141	ns
**KIR**							
-3DL1/3DL1	15 (63)	2 (25)	13 (81)	0.05	0.004–0.6	**0.017**	**0.011**
-3DL1/3DS1	9 (38)	6 (75)	3 (18)	20.8	1.7–250	**0.017**	**0.011**
-3DS1/3DS1	0 (0)	0 (0)	0 (0)	1	—	—	ns
**HLA Ligands**							
-Bw4	21 (88)	6 (75)	15 (94)	0.50	0.03–6.2	0.565	ns
-Bw6	12 (50)	7 (88)	5 (31)	12.2	1.4–112	**0.027**	**ns**
-Bw4_Bw4	12 (50)	1 (13)	11 (69)	0.08	0.01–0.8	**0.027**	**ns**
-Bw4_Bw6	9 (38)	5 (63)	4 (25)	7.2	1.1–46.3	**0.038**	ns
-Bw6_Bw6	3 (13)	2 (25)	1 (6)	2.1	0.16–28.7	0.565	ns
**KIR+HLA-Ligand**							
-3DL1_Bw4	21 (88)	6 (75)	15 (94)	0.5	0.03–6.3	0.565	ns
-3DS1_Bw4	8 (33)	5 (63)	3 (19)	7.3	1.1–47.6	**0.037**	**ns**

Values are absolute with percentages in parentheses.

HCV; Hepatitis C virus, HLA; Human leucocyte antigen, KIR; Killer-cell immunoglobulin-like-receptors, OR; Odds Ratio, CI; Confidence interval, Pc, Benjamini-Yekutieli correction, ns; Non-significant.

#### 3.3. KIR-HLA associations

3.3.1. KIR-HLA-B: Statistically significant differences related to chronicity were only found in genetic factors present in the mother. Homozygosity for KIR3DL1/KIR3DL1 favoured viral clearance in infected children (Table 5). However, there was an increased risk of chronification among the infected children of heterozygous mothers for KIR3DL1/KIR3DS1 (Table 5).

#### 3.4. Mother and child allele matching and chronification of HCV infection in the child

The chronicity of HCV infection is related to closer genetic matching between mother and child in the analysis of All-HLA, HLA-Class-I and HLA-Class-II. In all cases, the genetic correlation between mother and child is stronger in children who remain chronically infected than in those who clear the virus (Table 6).

**Table 6:** HLA genomics, low resolution typing. Rates of mother/child allele matching and chronification of HCV in children.

	Chronic (n = 8)	Clearance (n = 16)	p-value
All-HLA*	67% ± 4.1	57% ± 1.2	**0.003**
HLA Class I*	58% ± 3.2	53% ± 1.7	0.075
HLA Class II*	73% ± 5.3	61% ± 1.9	0.729
HLA-A**			
** -Test- 50%**	7 (88)	16 (100)	0.257
** -Test+ 100%**	1 (13)	0 (0)	
HLA-B**			
** -Test- 50%**	6 (75)	14 (88)	0.421
** -Test+ 100%**	2 (25)	2 (13)	
HLA-Cw**			
** -Test- 50%**	5 (63)	15 (94)	0.089
** -Test+ 100%**	3 (38)	1 (6)	
HLA-DRB1**			
** -Test- 50%**	3 (38)	14 (88)	**0.009**
** -Test+ 100%**	6 (63)	2 (13)	
HLA-DQA1**			
** -Test- 50%**	3 (38)	13 (81)	**0.020**
** -Test+ 100%**	5 (63)	3 (19)	
HLA-DQB1**			
** -Test- 50%**	5 (63)	13 (81)	0.181
** -Test+ 100%**	3 (38)	3 (19)	
HLA-DPA1**			
** -Test- 50%**	0 (0)	5 (31)	0.189
** -Test+ 100%**	8 (100)	11 (69)	
HLA-DPB1**			
** -Test- 50%**	4 (50)	10 (67)	0.172
** -Test+ 100%**	4 (50)	5 (33)	
KIR*	84 ± 4.0	85 ± 3.5	0.966

HCV; Hepatitis C virus. HLA; Human leucocyte antigen

* Quantitative variables: Values are mean ± the standard error of the mean (SEM). Mann-Whitney test.

** Qualitative variables: Values are absolute with percentages in parentheses. Chi square test.

**Fig 1:**
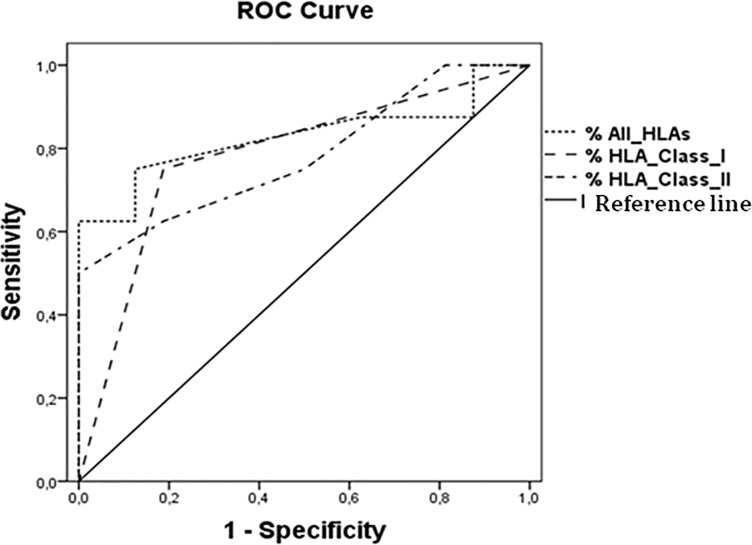
Receiver operating characteristic (ROC) curve of different HLA classifications (All-HLA, HLA class I and HLA class II) in mother and child matching and chronification of HCV infection in the child. ROC curves for % All-HLAs (area under the curve (AUC) = 0.83), % HLA class I (AUC = 0.78), % HLA class II (AUC = 0.78).

Receiver-operating characteristic (ROC) curves were used to determine the allele matches (Fig 1). The following values were obtained for the areas under the curve (AUC): All-HLA: AUC, 0.828; HLA-Class-I: AUC, 0.781; HLA-Class-II: AUC, 0.781.

If allele matching is considered to be an indicator of HCV chronicity in the child, its determination via “All-HLA” can be termed a good prognostic test, because an area of 0.828 was obtained under the curve. The best cut-off point was 65.6%, which corresponds to the allele match. The sensitivity obtained was 75% and the specificity, 87%. PPV = 74% and NPV = 87%. In other words, for “All-HLA”, among all the infected children included in the study whose allele matching with the mother exceeds 65.6%, 74% of them will become chronically ill. In the case of HLA-Class-I, of the infected children whose allele matching with the mother exceeds 58.4%, 65% will become chronically ill. Finally, for HLA-Class-II, among the infected children whose allele matching with the mother exceeds 75%, 62% will become chronically ill. Therefore, in this case the best test would be the one in which all HLA alleles are taken into consideration.

In the prognostic test of individual alleles, because the child may have only 50% matching with the mother or may have 100%, a test is considered positive when the matching is 100% and negative when it is 50%. Therefore, ROC curves cannot be used, and contingency tables must be applied. In our analysis, 100% matching was considered a positive test, and 50% matching was considered a negative one. The largest allele matching differences with the mother, for chronically infected children and those who had cleared the virus, were recorded for DRB1 and DQA1 (Table 6). Sensitivity and specificity were calculated from the same contingency tables. Therefore, it can be said that for DRB1, of all the infected children whose allele matching with their mother is 100%, 63% will become chronically ill. For DQA1, the corresponding figure is also 63% (Table 6).

## Discussion

This study analyses the role of various genetic factors–HLA, KIR receptors and the association between KIRs and HLA-Class I ligands–in relation to the MTCT of HCV and the chronicity of the disease in children. Both KIR receptors and their ligands have been associated with immune-inflammatory diseases[14], various types of tumour[15,16] and even viral infections[17–20]. Although questions related to HIV co-infection have been analysed extensively[21], our understanding of MTCT and the chronicity of HCV remains incomplete.

A wide variety of HLA alleles (class I and class II), in the mother and the child, have been shown to be related to MTCT. However, these data may be misleading, as HLA are highly polymorphic. Hence, statistical corrections may be needed in order to present an accurate result, and in such cases the Benjamini-Yekutieli correction is normally applied. A study in this field concluded that the DRB1*04 allele is a protective factor against vertical transmission, which was found to be more frequent among mothers who had not transmitted HCV infection to their children. However, in the latter study no statistical correction was performed [22]. In our own study, exactly the same result was obtained for the DRB1*04 (P = 0.06) but when the correction was applied, the statistical significance was lost.

KIR receptors can function either independently, due to their capability to activate or inhibit the immune response, or when bound to their HLA ligands. This binding can be analysed when the ligand is known and is present in a subject. The study of HLA ligands shows there is an association between MTCT and type C ligands. In particular, in mothers the presence of the HLA-C1 ligand is a protective factor against HCV infection, while if the ligand is of the HLA-C2C2 type, there is a greater probability of the infection being transmitted to the child. Our analysis of the association between the KIRs and their HLA ligands shows that when a KIR receptor binds to an HLA-C1 ligand, as in the case of KIR2DS4-C1, there is a protective effect against infection, but with P correction it is not significant. Moreover, according to our multivariate analysis, the HLA-C1 ligand is a factor that is independently protective against MTCT. As KIR2DL3 is statistically significant in itself, we assume that the association between the KIR and its ligand would further enhance protection against HCV infection. These important findings have been described previously with respect to HCV infection, as the HLA-C1-KIR2DL3 combination is associated with the spontaneous elimination of the infection. To explain this, it has been hypothesised that the bond between KIR2DL3 and HLA-C1 presents a weaker physical and chemical affinity than the bond with other receptors such as KIR2DL1 and KIR2DL2[23]. Such differences in affinity would reduce NK inhibition, favouring their activation and thus the control of infection. With regard to the possibilities of chronic HCV infection, it has been shown that the DQA1*01 allele present in mothers is related to the chronification of the disease in children. Conversely, the DQB1*03 present in the child favours viral clearance. Both of these HLAs are class II. This finding is consistent with previous reports according to which this allele is a protective factor, and not only DQB1*03 but also DQB1*03:01 or 03:02[24–26]. In previous studies by our own group[27] and by other authors[28], this allele has been associated with sustained virologic response (SVR) to treatment with pegIFN/RBV[27]. With respect to KIRs, in our study the presence of both KIR2DS1 and KIR3DS1 in the mother favoured viral persistence, although Rivero-Juarez et al.[28] related KIR3DS1 with viral clearance. This discrepancy may be explained by the fact that the latter study was conducted in patients who were HIV/HCV co-infected, and such patients were excluded from our study population. Furthermore, in children, KIR2DS3 favours viral clearance, a finding that conflicts with the data reported by Dring et al.[29] for a cohort of Irish patients. However, these authors, unlike the present study, did not consider infected children but rather populations of adults who had been diagnosed previously. By types of HLA ligand, the chronification of the virus in children is more strongly related to HLA-B than HLA-C[29]. Thus, our data show that the children of mothers whose ligand is type HLA-Bw6 are 12.2 times more likely to become chronically infected; however, if the ligand is type HLA-Bw4/HLA-Bw4 it is protective against chronification of the virus in the children. This ligand has been linked to SVR against HCV[17], but we were unable to find any bibliographic data on MTCT or chronification in the child. The effect of HLA-Bw4 can be observed in the presence of homozygous KIR3DL1/KIR3DL1 in the mother. However, heterozygous KIR3DL1/KIR3DS1 and the binding of HLA-Bw4 KIR3DS1 both favour chronification of the virus in children. Thus, while homozygous HLA-Bw4 is protective, the effect of KIR3SD1 is more powerful than that of HLA and so the former prevails, favouring persistence of the virus. These findings are consistent with those of the previous study on the effect of KIRs. These data continue to be of great interest even when application of the Benjamini-Yekutieli correction eliminates the statistical significance. In addition, the statistical power of our results should be taken into account, since all the data were corrected for IL28B and viral load.

In the cohort of HCV-chronic mothers, it was hypothesised that the greater the immune-genetic similarity between mother and child, the greater the likelihood of chronification in the child. Regarding MTCT, no differences related to allele matching have been reported. In our chronification study, statistically significant differences were observed in the rate of mother/child allele matching, for all HLAs (P = 0.003). For KIRs, no such differences were found. When each HLA is analysed separately, the differences are mainly due to two HLA-Class II alleles: HLA-DRB1 (P = 0.009) and HLA-DQA1 (P = 0.02). By making use of ROC curves, these data can be applied as a possible prognostic test, thanks to the high levels of sensitivity and specificity obtained and the good PPV (74%) and NPV (87%) values.

Two hypotheses are proposed: 1) when maternal mononuclear cells (MNC) are infected with HCV and passed to the child, they may express surface HLA molecules that are different from those of the child; in consequence, the child’s immune system seeks to eliminate them, with consequent viral destruction. However, when there is greater HLA matching, these cells survive for longer within the child, facilitating infection. This theory is substantiated by the findings of Azzari et al. [30], who concluded that maternal MNC infection is associated with HCV-VT and, moreover, that maternal cells survive for longer in their children when mother and child present identical HLA in certain alleles. 2) The selective pressure exerted by the maternal immune response to the virus (T-cell response mediated by HLA), generates a series of viral variants (quasispecies) that can elude this immune response and be transmitted to the child; therefore, children with HLA matching that of the mother present quasispecies that are capable of eluding the immune system and thus are at greater risk of infection. The importance of positive MNC in the mother’s peripheral blood and/or breast milk has been highlighted previously[6]

In summary, the genetic factors of mothers and newborns should be studied in order to understand the processes by which MTCT takes place and the likelihood of chronic infection in the child. It has been shown that the genetic factors related to MTCT are different from those which facilitate chronic infection in children. We also know that the degree of genetic matching between mother and child is of major importance: the more similar they are in this respect, the less likely that the child will clear the virus spontaneously. This relationship is based on the fact that when a mother presents certain immunological characteristics that fail to clear HCV, if her child is very similar genetically, then a similar response to infection should be expected. Therefore, it is the immune system itself that prevents the organism from responding properly. In our study population, this relationship was apparent regarding the chronification of the disease in the child. Also relevant to this understanding is the fact that allele matching between mother and child is an important indicator of the evolution of the disease in the child, although this factor has no relation to the vertical transmission of the virus. The study of these genetic factors could guide the future treatment of pregnant women with increased risk of transmission of the virus to their children or acute infection in infants. These findings imply that NK cells may play a previously unappreciated role in protecting against MTCT and that both NK cell immunity and adaptive T-cell responses may influence viral clearance in infected children.

## Supporting information

S1 Table Genetic variability in RNA-HCV(+) mothers and their children. (PDF)

S2 Table KIR frequencies. (PDF)

S3 Table MTCT results. (PDF)

S4 Table Chronification results. (PDF)

## Abbreviations

HLA: human leucocyte antigen
KIR: killer-cell immunoglobulin-like receptor
HCV: hepatitis C virus
RNA: Ribonucleic acid
MTCT: mother-to-child transmission
HIV: human immunodeficiency virus
NK: natural killer cell
ALT: alanine aminotransferase
DNA: deoxyribonuclic acid
SSO: sequence-specific oligonucleotide
RSSO: reverse sequence-specific oligonucleotide
PCR: Polymerase chain reaction
OR: odds ratio
CI: confidence interval
SEM: standard error of the mean
ROC: receiver operating characteristic
PPV: positive predictive value
NPV: negative predictive value
SVR: sustained virologic response
RBV: ribavirin
