# Supplementary material for: Influence of HLA class I, HLA class II and KIRs on vertical transmission and chronicity of hepatitis C virus in children
Source: PLoS One. 2017 Feb 22;12(2):e0172527. doi: 10.1371/journal.pone.0172527 (PMC5321427; doi:10.1371/journal.pone.0172527)
Supplement: S1 Table — (PDF) [file pone.0172527.s001.pdf]

S1 Table

HLA High Resolution				
**HLA**	**A**	**B**	**C**	**DRB1**
**Mother, (n=79)**	19 (24)	36 (46)	19 (24)	23 (29)
**Child, (n=98)**	19 (19)	32 (33)	20 (20)	24 (24)

**HLA**	**DQA1**	**DQB1**	**DPA1**	**DPB1**
**Mother, (n=79)**	10 (13)	15 (19)	5 (6)	15 (19)
**Child, (n=98)**	11 (11)	16 (16)	4 (4)	16 (16)

HLA Low Resolution				
**HLA**	**A**	**B**	**C**	**DRB1**
**Mother, (n=79)**	16 (20)	23 (29)	13 (81)	11 (69)
**Child, (n=98)**	15 (15)	22 (22)	13 (13)	11 (73)

**HLA**	**DQA1**	**DQB1**	**DPA1**	**DPB1**
**Mother, (n=79)**	5 (6)	5 (6)	4 (5)	13 (17)
**Child, (n=98)**	6 (6)	5 (5)	3 (3)	14 (14)

Values are absolute with percentages in parentheses

HCV; Hepatitis C virus, HLA; Human leucocyte antigen
