# Supplementary material for: Influence of HLA class I, HLA class II and KIRs on vertical transmission and chronicity of hepatitis C virus in children
Source: PLoS One. 2017 Feb 22;12(2):e0172527. doi: 10.1371/journal.pone.0172527 (PMC5321427; doi:10.1371/journal.pone.0172527)
Supplement: S2 Table — (PDF) [file pone.0172527.s002.pdf]

S2 Table

KIR	KIR2DL1	KIR2DL2	KIR2DL3	KIR2DL4
**Mother (n=79)**	77 (98)	44 (56)	71 (90)	79 (100)
**Child (n=98)**	95 (97)	51 (52)	93 (95)	98 (100)
**KIR**	**KIR2DL5**	**KIR2DP1**	**KIR2DS1**	**KIR2DS2**
**Mother (n=79)**	31 (39)	79 (100)	24 (30)	42 (53)
**Child (n=98)**	49 (50)	95 (97)	37 (38)	49 (50)
**KIR**	**KIR2DS3**	**KIR2DS4**	**KIR2DS5**	**KIR3DL1**
**Mother (n=79)**	19 (24)	78 (99)	16 (20)	78 (99)
**Child (n=98)**	27 (28)	92 (94)	28 (29)	91 (93)
**KIR**	**KIR3DL2**	**KIR3DL3**	**KIR3DP1**	**KIR3DS1**
**Mother (n=79)**	79 (100)	79 (100)	79 (100)	26 (33)
**Child (n=98)**	98 (100)	98 (100)	98 (100)	41 (42)

Values are absolute with percentages in parentheses.
KIR; killer-cell immunoglobulin-like receptors.
