# Supplementary material for: Influence of HLA class I, HLA class II and KIRs on vertical transmission and chronicity of hepatitis C virus in children
Source: PLoS One. 2017 Feb 22;12(2):e0172527. doi: 10.1371/journal.pone.0172527 (PMC5321427; doi:10.1371/journal.pone.0172527)
Supplement: S3 Table — (PDF) [file pone.0172527.s003.pdf]

S3 Table

MOTHER-TO-CHILD TRANSMISSION						
**MOTHERS**						
**HAPLOTYPES**	**ALL (n=98)**	**YES (n=24)**	**NO (n=74)**	**ORc**	**CI**	***p-value***
**A**	98 (100)	24 (100)	74 (100)	---	---	---
**B**	68 (69)	18 (75)	51 (68)	---	---	ns
**AA**	30 (31)	6 (25)	24 (32)	---	---	ns
**AB**	68 (69)	18 (75)	50 (68)	---	---	ns
**BB**	0 (0)	0 (0)	0 (0)	---	---	---
**AA**	30 (31)	6 (25)	24 (32)	---	---	ns
**Bx**	68 (69)	18 (75)	50 (68)	---	---	ns
**HLA Ligands Type C and KIRs**	**ALL (n=98)**	**YES (n=24)**	**NO (n=74)**	**ORc**	**CI**	***p-value***
**C1C2**	47 (49)	12 (50)	35 (49)	---	---	ns
**2DL2_2DL2**	9 (9)	2 (9)	7 (9)	---	---	ns
**2DL2_2DL3**	45 (46)	13 (57)	32 (43)	---	---	ns
**2DL3_3DL3**	43 (44)	8 (35)	35 (47)	---	---	ns
**2DL2/2DL2-C1C1**	2 (2)	0 (0)	2 (3)	---	---	ns
**2DL2/2DL2-C1C2**	6 (6)	1 (14)	5 (7)	---	---	ns
**2DL2/2DL2-C2C2**	1 (1)	1 (4)	0 (0)	---	---	ns
**2DL2/2DL3-C1C1**	11 (12)	1 (4)	10 (14)	---	---	ns
**2DL2/2DL3-C1C2**	29 (31)	9 (39)	20 (28)	---	---	ns
**2DL3/2DL3-C1C1**	15 (16)	1 (4)	14 (19)	---	---	ns
**2DL3/2DL3-C1C2**	11 (12)	1 (4)	10 (14)	---	---	ns
**2DL3/2DL3-C2C2**	16 (17)	6 (26)	10 (14)	---	---	ns
**2DL2-C1**	48 (51)	11 (48)	37 (51)			ns
**2DS1_C2**	21 (22)	8 (33)	13 (18)			ns
**2DS2_C1**	44 (46)	11 (46)	33 (46)			ns
**2DS3_C1**	22 (23)	6 (25)	16 (22)			ns
**HLA Ligands Type B and KIRs**	**ALL (n=98)**	**YES (n=24)**	**NO (n=74)**	**ORc**	**CI**	***p-value***
**Bw4**	86 (88)	21 (88)	65 (88)	---	---	ns
**Bw6**	62 (63)	12 (50)	50 (68)	---	---	ns
**Bw4_Bw4**	36 (37)	12 (50)	24 (32)	---	---	ns
**Bw4_Bw6**	50 (51)	9 (38)	41 (55)	---	---	ns
**Bw6_Bw6**	12 (12)	3 (13)	9 (12)	---	---	ns
**3DL1/3DL1**	64 (65)	15 (63)	49 (66)	---	---	ns
**3DL1/3DS1**	33 (33)	9 (38)	24 (32)	---	---	ns
**3DS1/3DS1**	1 (1)	0 (0)	1 (1)	---	---	ns
**3DL1_Bw4**	85 (87)	21 (88)	64 (87)	---	---	ns
**3DS1_Bw4**	31 (31)	8 (33)	23 (31)	**---**	**---**	ns

CHILDREN						
**HAPLOTYPES**	**ALL (n=98)**	**YES (n=24)**	**NO (n=74)**	**OR**	**CI**	***p-value***
**A**	98 (100)	24 (100)	74 (100)	---	---	---
**B**	70 (72)	16 (67)	54 (73)	---	---	ns
**AA**	28 (28)	8 (33)	20 (27)	---	---	ns
**AB**	70 (71)	16 (67)	55 (73)	---	---	ns
**BB**	0 (0)	0 (0)	0 (0)	---	---	---
**AA**	28 (28)	8 (33)	20 (27)	---	---	ns
**Bx**	70 (71)	16 (67)	55 (73)	---	---	ns
**HLA Ligands Type C and KIRs**	**ALL (N=96)**	**YES (n=24)**	**NO (n=72)**	**OR#**	**CI**	***p-value***
**C2** (C2C2, C1C2)	73 (76)	19 (79)	54 (75)	---	---	ns
**C1C1**	23 (24)	5 (21)	18 (25)	---	---	ns
**C1C2**	58 (60)	12 (50)	46 (64)	---	---	ns
**2DL3_3DL3**	47 (48)	13 (54)	34 (46)	---	---	ns
**2DL2/2DL2-C1C1**	1 (1)	1 (4)	0 (0)	---	---	ns
**2DL2/2DL2-C1C2**	2 (2)	1 (4)	1 (1)	---	---	ns
**2DL2/2DL3-C1C1**	11 (12)	3 (13)	8 (11)	---	---	ns
**2DL2/2DL3-C2C2**	8 (7)	1 (4)	7 (10)	---	---	ns
**2DL3/2DL3-C1C1**	11 (12)	1 (4)	10 (14)	---	---	ns
**2DL3/2DL3-C1C2**	30 (31)	8 (33)	22 (31)	---	---	ns
**2DL1-C2**	70 (74)	17 (74)	53 (74)			ns
**2DL2-C1**	40 (41)	8 (33)	32 (43)			ns
**2DS1_C2**	23 (24)	7 (29)	16 (22)			ns
**2DS2_C1**	38 (39)	8 (33)	30 (41)			ns
**2DS3_C1**	23 (24)	6 (25)	17 (23)			ns
**2DS4_C1**	75 (78)	16 (67)	59 (82)			ns
**2DS4_C2**	70 (73)	18 (75)	52 (72)			ns
**HLA Ligands Type B and KIRs**	**ALL (N=98)**	**YES (n=24)**	**NO (n=74)**	**ORc**	**CI**	***p-value***
**Bw4**	82 (84)	20 (83)	62 (84)	---	---	ns
**Bw6**	65 (66)	15 (63)	50 (68)	---	---	ns
**Bw4_Bw4**	33 (34)	9 (38)	24 (32)	---	---	ns
**Bw4_Bw6**	49 (50)	11 (46)	38 (51)	---	---	ns
**Bw6_Bw6**	16 (16)	4 (17)	12 (16)	---	---	ns
**3DL1/3DL1**	57 (58)	12 (50)	45 (60)	---	---	ns
**3DL1/3DS1**	34 (35)	11 (46)	24 (31)	---	---	ns
**3DS1/3DS1**	7 (7)	1 (4)	6 (8)	---	---	ns
**3DL1_Bw4**	75 (76)	19 (79)	56 (75)	---	---	ns
**3DS1_Bw4**	33 (33)	9 (38)	24 (32)	---	---	ns

Values are absolute with percentages in parentheses.
MTCT; Mother-to-child transmission, HCV; Hepatitis C virus, HLA; Human leucocyte antigen, KIR; Killer-cell immunoglobulin-like receptors, OR; Odds Ratio, CI; Confidence interval, ns; Not significant
