# Supplementary material for: Influence of HLA class I, HLA class II and KIRs on vertical transmission and chronicity of hepatitis C virus in children
Source: PLoS One. 2017 Feb 22;12(2):e0172527. doi: 10.1371/journal.pone.0172527 (PMC5321427; doi:10.1371/journal.pone.0172527)
Supplement: S4 Table — (PDF) [file pone.0172527.s004.pdf]

S4 Table

CHRONIFICATION IN CHILDREN MOTHERS						
**HAPLOTYPES MOTHERS**	**ALL (n=24)**	**YES (n=8)**	**NO (n=16)**	**OR#**	**CI**	***p-value***
**A**	24 (100)	8 (100)	16 (100)	---	---	---
**BB**	0 (0)	0 (0)	0 (0)	---	---	---
**HLA Ligands Type C and KIRs**	**ALL (n=24)**	**YES (n=8)**	**NO (n=16)**	**OR**	**CI**	***p-value***
**C1** (C1C1, C1C2)	14 (58)	5 (63)	9 (56)	---	---	ns
**C2** (C2C2, C1C2)	22 (92)	8 (100)	14 (88)	---	---	ns
**C1C1**	2 (8)	0 (0)	2 (13)	---	---	ns
**C1C2**	12 (50)	5 (63)	7 (44)	---	---	ns
**C2C2**	10 (42)	3 (38)	7 (44)	---	---	ns
**2DL2_2DL3**	2 (9)	1 (14)	1 (6)	---	---	ns
**2DL2_2DL3**	13 (56)	4 (57)	9 (56)	---	---	ns
**2DL3_3DL3**	8 (35)	2 (29)	6 (38)	---	---	ns
**2DL2/2DL2-C1C1**	0 (0)	0(0)	0 (0)	---	---	ns
**2DL2/2DL2-C1C2**	1 (4)	0 (0)	1 (6)	---	---	ns
**2DL2/2DL2-C2C2**	1 (4)	1 (14)	0 (0)	---	---	ns
**2DL2/2DL3-C1C1**	1 (4)	0 (0)	1 (6)	---	---	ns
**2DL2/2DL3-C1C2**	9 (39)	4 (57)	5 (31)	---	---	ns
**2DL2/2DL3-C2C2**	3 (13)	0 (0)	3 (19)	---	---	ns
**2DL3/2DL3-C1C1**	1 (4)	0 (0)	1 (6)	---	---	ns
**2DL3/2DL3-C1C2**	1 (4)	0 (0)	1 (6)	---	---	ns
**2DL3/2DL3-C2C2**	6 (26)	2 (29)	4 (25)	---	---	ns
**2DL1-C2**	22 (92)	8 (100)	14 (88)	---	---	ns
**2DL2-C1**	11 (48)	4 (57)	7 (44)	---	---	ns
**2DL3-C1**	13 (54)	5 (63)	8 (50)	---	---	ns
**2DS2_C1**	11 (46)	4 (50)	7 (44)	---	---	ns
**2DS3_C1**	6 (25)	2 (25)	4 (25)	---	---	ns
**2DS4_C1**	14 (58)	5 (63)	9 (56)	---	---	ns
**2DS4_C2**	22 (92)	8 (100)	14 (88)	---	---	ns
**HLA Ligands Type B and KIRs**	**ALL (n=98)**	**YES (n=24)**	**NO (n=74)**	**OR**	**CI**	***p-value***
**Bw4**	21 (88)	6 (75)	15 (94)	---	---	ns
**Bw4_Bw6**	9 (38)	5 (63)	4 (25)	---	---	ns
**Bw6_Bw6**	3 (13)	2 (25)	1 (6)	---	---	ns
**3DS1/3DS1**	0 (0)	0 (0)	0 (0)	---	---	---
**3DL1_Bw4**	21 (88)	6 (75)	15 (94)	---	---	ns

CHILDREN						
**HAPLOTYPES**	**ALL (n=24)**	**YES (n=8)**	**NO (n=16)**	**OR**	**CI**	***p-value***
**A**	24 (100)	8 (100)	16 (100)	---	---	---

**B**	16 (67)	5 (63)	11 (69)	---	---	ns
**AA**	8 (33)	3 (38)	5 (31)	---	---	ns
**AB**	16 (67)	5 (63)	11 (69)	---	---	ns
**BB**	0 (0)	0 (0)	0 (0)	---	---	---
**AA**	8 (33)	3 (38)	5 (31)	---	---	ns
**Bx**	16 (67)	5 (63)	11 (69)	---	---	ns
**HLA Ligands Type C and KIRs**	**ALL (n=24)**	**YES (n=8)**	**NO (n=16)**	**OR**	**CI**	***p-value***
**C1** (C1C1, C1C2)	17 (71)	7 (88)	10 (63)	---	---	ns
**C2** (C2C2, C1C2)	19 (79)	7 (88)	12 (75)	---	---	ns
**C1C1**	5 (21)	1 (13)	4 (25)	---	---	ns
**C1C2**	12 (50)	6 (75)	6 (38)	---	---	ns
**C2C2**	7 (29)	1 (13)	6 (38)	---	---	ns
**2DL2_2DL3**	4 (17)	1 (13)	3 (19)	---	---	ns
**2DL2_2DL3**	7 (29)	2 (25)	5 (31)	---	---	ns
**2DL3_3DL3**	13 (54)	5 (63)	8 (50)	---	---	ns
**2DL2/2DL2-C1C1**	1 (4)	0 (0)	1 (6)	---	---	ns
**2DL2/2DL2-C1C2**	1 (4)	1 (13)	0 (0)	---	---	ns
**2DL2/2DL2-C2C2**	2 (8)	0 (0)	2 (13)	---	---	ns
**2DL2/2DL3-C1C1**	3 (13)	1 (13)	2 (13)	---	---	ns
**2DL2/2DL3-C1C2**	3 (13)	1 (13)	2 (13)	---	---	ns
**2DL2/2DL3-C2C2**	1 (4)	0 (0)	1 (6)	---	---	ns
**2DL3/2DL3-C1C1**	1 (4)	0 (0)	1 (6)	---	---	ns
**2DL3/2DL3-C1C2**	8 (33)	4 (50)	4 (25)	---	---	ns
**2DL3/2DL3-C2C2**	4 (17)	1 (13)	3 (19)	---	---	ns
**2DL1-C2**	17 (74)	6 (75)	11 (73)	---	---	ns
**2DL2-C1**	8 (33)	2 (25)	6 (37)	---	---	ns
**2DL3-C1**	15 (63)	6 (75)	9 (56)	---	---	ns
**2DS1_C2**	7 (29)	2 (25)	5 (31)	---	---	ns
**2DS2_C1**	8 (33)	3 (38)	5 (31)	---	---	ns
**2DS4_C1**	16 (67)	6 (75)	10 (63)	---	---	ns
**2DS4_C2**	18 (75)	6 (75)	12 (75)	---	---	ns
**HLA Ligands Type B and KIRs**	**ALL (n=24)**	**YES (n=8)**	**NO (n=16)**	**OR**	**CI**	***p-value***
**Bw4**	20 (83)	6 (75)	14 (88)	---	---	ns
**Bw6**	15 (63)	6 (75)	9 (56)	---	---	ns
**Bw4_Bw4**	9 (38)	2 (25)	7 (44)	---	---	ns
**Bw4_Bw6**	11 (46)	4 (50)	7 (44)	---	---	ns
**Bw6_Bw6**	4 (17)	2 (25)	2 (13)	---	---	ns
**3DL1/3DL1**	12 (50)	4 (50)	8 (50)	---	---	ns
**3DL1/3DS1**	11 (46)	3 (38)	8 (50)	---	---	ns
**3DS1/3DS1**	1 (4)	1 (13)	0 (0)	---	---	ns
**3DL1_Bw4**	19 (79)	5 (63)	14 (88)	---	---	ns
**3DS1_Bw4**	9 (38)	3 (38)	6 (38)	---	---	ns

Values are absolute with percentages in parentheses.
MTCT; Mother-to-child transmission, HCV; Hepatitis C virus, HLA; Human leucocyte antigen, KIR; Killer-cell immunoglobulin-like receptors, OR; Odds Ratio, CI; Confidence interval, ns; Not significant
